# Fetal hypoplastic left heart syndrome and maternal liver transplantation for Wilson’s disease: a case report

**DOI:** 10.1186/1752-1947-7-276

**Published:** 2013-12-30

**Authors:** Antonia Wenners, Colin Petko, Constantin von Kaisenberg, Alexander Strauss, Christel Eckmann-Scholz, Ulrike Hoffmann, Walter Jonat, Ibrahim Alkatout

**Affiliations:** 1 Department of Gynecology and Obstetrics, University Hospitals Schleswig-Holstein, Campus Kiel, Arnold-Heller Strasse 3, Building 24, 24105 Kiel, Germany; 2 Department of Congenital Heart Disease and Pediatric Cardiology, University Hospitals Schleswig-Holstein, Campus Kiel, 24105 Kiel, Germany; 3 Department of Obstetrics, Gynecology and Reproductive Medicine, Hannover Medical School, Hannover, Germany

**Keywords:** Hypoplastic left heart syndrome, Immunosuppression, Liver transplantation, Malformation, Teratogenicity, Wilson’s disease

## Abstract

**Introduction:**

Liver transplantation currently represents the only curative treatment for Wilson’s disease. A lifelong immunosuppressive therapy is mandatory. In spite of increased maternal and fetal risks, pregnancies after liver transplantation have been reported with favorable perinatal outcomes. Hypoplastic left heart syndrome is a spectrum of congenital heart defects that results in the inability to support the systemic circulation. Although its etiology remains elusive, the prognosis of this previously fatal condition has dramatically improved over the last 2 decades mainly due to advances in prenatal diagnosis, surgical technique and perioperative care.

**Case presentation:**

We present a case of a Caucasian 26-year-old woman, gravida 2, para 1 at 36^+0^ weeks of gestation who had received a liver transplantation due to Wilson’s disease and subsequently delivered a child with hypoplastic left heart syndrome.

**Conclusions:**

This coincidence of medical conditions has not been described in the literature so far and its implications for mother and child as well as the pathophysiological mechanisms are discussed on the basis of a literature review.

## Introduction

Several acquired, inherited or autoimmune hepatic disorders can necessitate liver transplantation. Wilson’s disease is a rare autosomal recessive disorder of copper metabolism that can lead to hepatic failure and neurological degeneration. The underlying mechanism is a mutation of the copper-transporting adenosine triphosphatase gene (*ATP7B*) located on the long arm of chromosome 13. The worldwide prevalence is 1 in 30,000. Clinical manifestation due to copper accumulation is highly variable, ranging from mild hepatitis to acute liver failure and cirrhosis. Liver transplantation with lifelong immunosuppressive therapy represents the only curative treatment [1].

Hypoplastic left heart syndrome (HLHS) is a congenital heart defect characterized by variable hypoplasia of the left ventricle, stenosis or atresia of mitral and/or aortic valves and aortic arch hypoplasia, resulting in the inability of the left side of the heart to support the systemic circulation. This cardiac malformation occurs in about 0.02% of all live births. The etiology of HLHS is not yet fully understood but a multifactorial pathogenesis can be assumed. Risk factors might include familial, infectious, maternal and gestational conditions. A genetic influence seems probable although no specific gene has been identified so far. Whether maternal immunosuppression during pregnancy can cause HLHS is unknown [2,3].

The first pregnancy in a liver transplant recipient was reported by Walcott *et al*. in 1978 [4]. Since then, hundreds of pregnancies after orthotopic liver transplantation have been reported with favorable perinatal outcomes. We present a case of HLHS after maternal liver transplantation in adolescence due to Wilson’s disease and discuss the potential chain of cause and effect.

## Case presentation

A Caucasian 26-year-old woman, gravida 2, para 1 at 36^+0^ weeks of gestation was admitted to our hospital with fetal HLHS and contractions, not affecting her cervix. She had received a liver transplant at 16 years of age because of acute liver failure due to Wilson’s disease. Her immunosuppression was maintained with tacrolimus. Her first pregnancy at the age of 21 years had been uneventful.

**Figure 1:**
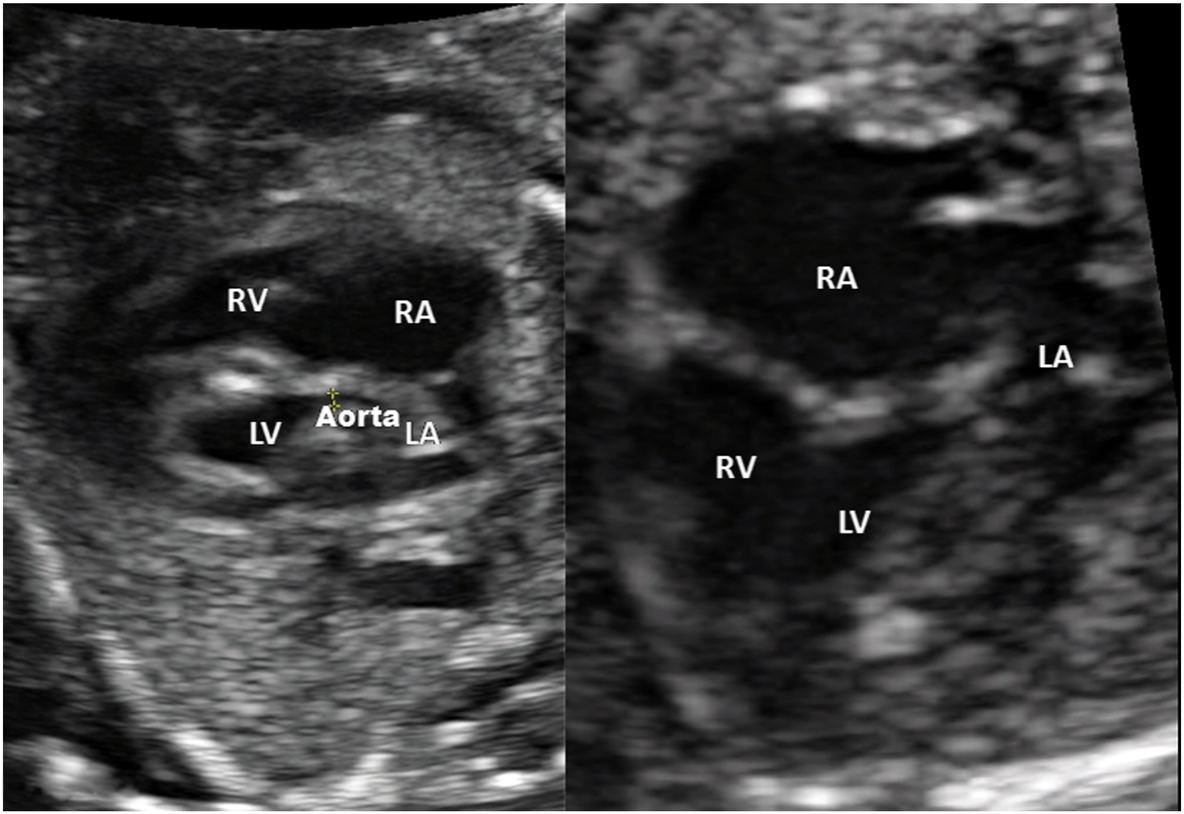
**Prenatal fetal echocardiography showing a four chamber view of the fetus affected with hypoplastic left heart syndrome.** Abbreviations: LA, Left atrium; LV, Left ventricle; RA, Right atrium; RV, Right ventricle.

In the second pregnancy a prenatal ultrasound scan at 18 weeks’ gestation revealed fetal HLHS (Figure 1). The fetus’s left ventricle and ascending aorta were hypoplastic (1.5 mm aortal width) and were perfused retrogradely via the ductus arteriosus. The fetus’s right ventricular function was adequate. In the medical history of the patient, no cardiac disease was known in the concerned families. The further pregnancy course was uneventful with normal maternal liver and renal function under immunosuppression with tacrolimus. The fetal growth, Doppler analysis and amniotic fluid were in normal ranges. No signs of preeclampsia, infection or transplant rejection were apparent.

Primary re-cesarean section was performed at 37^+0^ weeks of gestational age. A male neonate weighing 3380 g was delivered, his APGAR scores (the criteria are appearance, pulse, grimace, activity and respiration) were 8, 9 and 10, pH was 7.24 and base excess −2.8.

**Figure 2:**
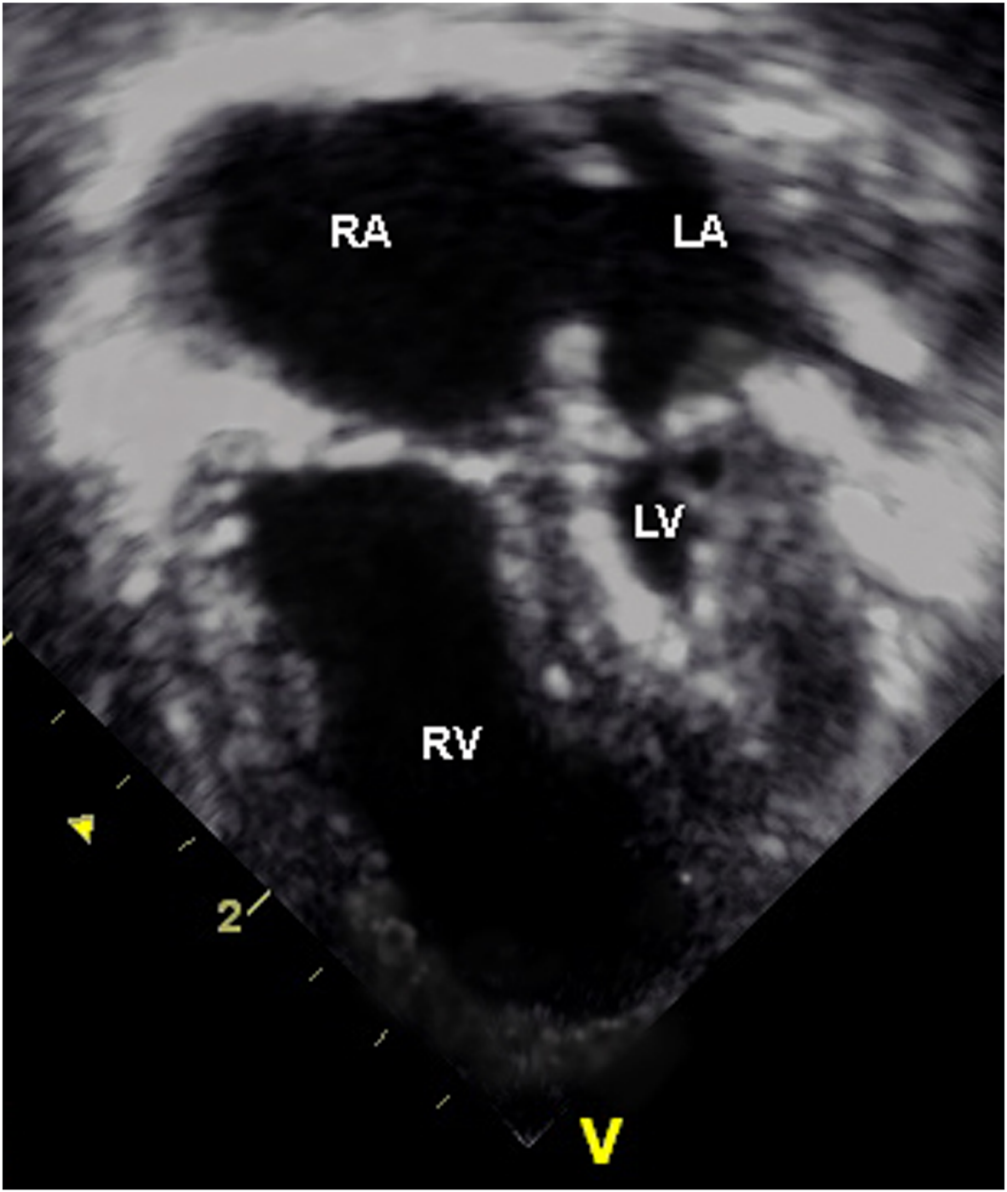
**Postnatal transthoracal echocardiography (four chamber view): severe hypoplasia of the left ventricle with endocardial fibroelastosis and hypoplasia of the left atrium are seen.** Abbreviations: LA, Left atrium; LV, Left ventricle; RA, Right atrium; RV, Right ventricle.

The newborn was set on prostaglandin infusion therapy. Postnatal echocardiography confirmed the diagnosis of HLHS with mitral and aortic valve atresia as well as hypoplasia of his left ventricle and ascending aorta (Figure 2). Other medication given to the newborn included intravenous furosemide and afterload reduction with sodium nitroprusside. A Norwood operation with a 3.5 mm Blalock–Taussig shunt was performed 3 days after delivery without complications. In the following weeks, digoxin and furosemide therapy was instituted orally to reduce symptoms of mild heart failure related to pulmonary over-circulation. He was on full oral feedings and was discharged from the hospital with a home monitoring protocol at the age of 6 weeks.

After delivery the mother was in perfect health, no rejection episode was observed and discharge was possible after 5 days. Immunosuppressive therapy was continued postnatally with tacrolimus adapted to serum blood levels.

## Discussion

We describe a case of HLHS after maternal liver transplantation due to Wilson’s disease. The most important risks for the fetus in mothers with a liver transplant are preterm delivery (31%) and low birth weight (23%). Attributed maternal risks are pregnancy-induced hypertension, preeclampsia and infection during pregnancy [5].

In patients who have had liver transplantation no significantly higher incidence of malformation patterns has been shown [5]. Abortions occur more often in transplant recipients whose underlying disease was autoimmune cirrhosis than in recipients with inherited disorders such as Wilson’s disease [5]. Wilson’s disease has not been associated with an increased risk for other genetic disorders.

Dei Malatesta *et al*. found that higher rates of both abortions (19%) and pregnancy complications in a transplantation collective were inversely related to the time interval between transplantation and conception [5]. While the timing of pregnancy is controversially discussed, current recommendations are to wait for at least 1 year after liver transplantation [6]. Despite the fact that the incidence of malformations is not higher in newborns after maternal liver transplantation compared to the general population, malformation patterns including severe heart defects could potentially account for the higher abortion rates [7].

The etiology of HLHS remains unknown but may include chromosomal, genetic, infectious, immunosuppressive, teratogenic conditions and other factors [3].

Although a genetic involvement is strongly suspected, no single gene has been identified to cause HLHS. Chromosomal anomalies occur in up to 5 to 12% of children with HLHS and an association with Turner’s syndrome as well as trisomy 18 and 13 has been described. The genetic alteration most commonly associated with HLHS is the terminal 11q deletion (Jacobsen’s syndrome) in which the incidence of HLHS is 10% [8]. The recurrence risk of HLHS is estimated to be 2 to 4% when a previous newborn was affected [8,9]. ATP7B is the gene product of the Wilson’s disease gene and is located on chromosome 13. More than 300 different mutations have been identified to date [10]. In Europe the most common mutation is the point mutation H1069Q in exon 14, which underlies 30 to 60% of all mutations in Caucasians [11]. A direct linkage between this gene defect in the mother and HLHS in the child seems to be not possible.

Increased rates of infection during pregnancy (20%) are known to occur in patients after liver transplantation compared to the general population. The higher incidence of infection during pregnancy in patients who have had liver transplantation is most probably related to immunosuppressant therapy. Table 1 shows that different immunosuppressants have a variable impact on the pregnancy course and outcome. Fetal exposure to active maternal infections including *Cytomegalovirus* (CMV) infection presents an increased risk for HLHS [3]. In our case, however, no infection was diagnosed during pregnancy; repeated testing for CMV was negative.

**Table 1:** Immunosuppressants and their side effects in pregnant liver transplant recipients

**Drug**	**Metabolism**	**Side effects**		**References**
		**In the mother**	**In the fetus**	
Corticosteroids	IL-2 synthesis inhibition	Hypertension Gestational diabetes Osteonecrosis Cataracts Striae	Malformation (4%, esp. cleft palate) Low birth weight PROM Adrenal suppression Infection	[6,12]
Cyclosporine	Inhibition of cytokine production	Hypertension Gestational diabetes Renal dysfunction Uterine dystonia Seizures	Malformation (3–5%; no consistent pattern known) Low birth weight Jaundice Cytopenia Hypoglycemia Intracerebral hemorrhage	[6,13,14]
Azathioprine	Antimetabolic: inhibition of the biosynthesis of purine nucleotides	Leukopenia Gastrointestinal side effects	Malformation (7%; esp. aphallia, hypo- or dysplasia of lungs and urinary bladder) IUGR Premature delivery Adrenal hypoplasia Infection	[6,13,15,16]
Tacrolimus	Inhibition of T-lymphocytes activation by inhibition of calcineurin	Hypertension Gestational diabetes Renal dysfunction Neurotoxicity Diarrhea Alopecia Pruritus	Malformation (6%; no consistent pattern known) Transient increase of potassium Impaired renal function	[6,17-19]
Mycophenolate mofetil	Inhibition of *de novo* synthesis of purines	Leukopenia Gastrointestinal side effects	Malformation (22%; esp. cleft lip and palate, micrognathia, ear malformations) 1st trimester fetal loss Anemia	[6,20,21]

*Abbreviations*: *esp*. especially, *IL* Interleukin, *IUGR* Intrauterine growth retardation, *PROM* Premature rupture of membranes.

Teratogenic agents can cause anatomical defects in the child, for example valproic acid. In terms of immunosuppression due to post-transplant prevention of graft rejection, potential benefits may outweigh these risks (Table 1). A person can also be immunosuppressed for a broad spectrum of other reasons, for example severe combined immunodeficiency or human immunodeficiency virus and rheumatic diseases. So far, no direct association between immunosuppressive treatment after liver transplantation and fetal heart malformation has been established. In particular, there is no evidence of an increased rate of fetal HLHS in immunosuppressed mothers. Although for tacrolimus the malformation rate is estimated to be 6%, several studies and reports confirm the safety of tacrolimus during pregnancy after transplantation [17-19].

Not a single case of HLHS has been described in connection with immunosuppression until now. We therefore feel that the likelihood of a causal relationship between tacrolimus therapy of the mother during pregnancy and HLHS in the fetus is low, in particular because the mother had delivered a healthy older child and had no history of abortions.

## Conclusions

In summary, liver transplantation affects pregnancy and fetal outcome, but an association with severe congenital heart defects or other malformations in newborns has not been established. However, it has to be considered that data may be limited due to underreporting or a small number of cases. The present case of the combination of fetal HLHS and maternal liver transplantation due to Wilson’s disease is probably coincidental, as there is little evidence in the literature for an underlying causal mechanism.

## Consent

Written informed consent was obtained from the patient for publication of this case report and accompanying images. A copy of the written consent is available for review by the Editor-in-Chief of this journal.

## Abbreviations

CMV: *Cytomegalovirus*; HLHS: Hypoplastic left heart syndrome.

## Competing interests

The authors of this manuscript have no conflicts of interest to disclose.

The manuscript was not prepared in any part by a commercial organization.

The manuscript was not funded in any part by a commercial organization, including educational grants.

## Authors’ contributions

AW and IA analyzed the case, reviewed the literature and wrote the manuscript. CP and UH gave data about the child’s course of disease and supplied the postpartal sonographic picture. CE, CK and AS supplied the prenatal data. AS and WJ analyzed the case, edited the manuscript and gave advice on many points. All authors read and approved the final manuscript.
